# Late Pleistocene faunal community patterns disrupted by Holocene human impacts

**DOI:** 10.1098/rsbl.2025.0151

**Published:** 2025-08-13

**Authors:** Barry W. Brook, S. Kathleen Lyons, Benjamin E. Carter, William Gearty, Orlin S. Todorov, Zach Aandahl, John Alroy

**Affiliations:** ^1^ School of Natural Sciences, University of Tasmania, Hobart, Tasmania 7001, Australia; ^2^ School of Biological Sciences, University of Nebraska-Lincoln, Lincoln, NE 68588, USA; ^3^ Department of Ecology and Evolutionary Biology, University of Michigan-Ann Arbor, Ann Arbor, MI 48109-1005, USA; ^4^ Open Source Program Office, Syracuse University, Syracuse, NY 13203, USA; ^5^ Tasmanian Institute of Agriculture, University of Tasmania, Launceston, Tasmania 7250, Australia; ^6^ School of Natural Sciences, Macquarie University, Sydney, New South Wales 2109, Australia

**Keywords:** biogeography, mammal communities, domestication, agriculture, faunal turnover, clustering, zooarchaeology, Holocene

## Abstract

We analysed fossil mammal assemblages from over 350 Late Pleistocene and Holocene sites worldwide to test whether human activities, such as agriculture, domestication and intensified land use, restructured global patterns of mammal co-occurrence. Using presence–absence data, we contrasted a novel iterative ‘chase clustering’ method, which is compositionally driven, against a traditional spatially constrained Ward’s clustering approach. Both methods recovered continental-scale groupings in the Pleistocene, consistent with known biogeographic boundaries. Holocene land use and domestication reconfigured these historical patterns, creating novel assemblages independent of previous biogeographic constraints. Faunal turnover at the local scale varied substantially across regions, being especially pronounced in the Americas, whereas other areas showed relative stability. Even moderate expansion of domesticates altered how communities grouped, highlighting their disproportionate ecological influence. Our findings demonstrate that human-driven niche modification, beyond earlier megafaunal extinctions, profoundly reshaped mammal communities on a global scale. Recognizing these anthropogenic legacies provides essential context for anticipating how current and future human pressures might further transform biodiversity.

## 1. Background

Human land use (burning, clearance, hunting) has shaped ecosystems for millennia; subsequent technological intensification further accelerated that influence [1]. As the Late Pleistocene transitioned into the Holocene, people refined hunting strategies and began cultivating crops, ushering in a new era of landscape modification [2,3]. Domestication soon followed, reorganizing ecosystems and reducing biodiversity and ecological complexity by favouring a handful of livestock and crop species over once-dominant wild taxa [4]. Although much attention has focused on the extinction of large-bodied ‘megafauna’ in the late Quaternary on multiple continents and islands [5,6], humans continued to reshape habitats in other ways, often through clearing forests, irrigation and converting wildlands into farmland or pasture [7].

These ecological transformations did not unfold uniformly in location or magnitude. Some regions lost iconic megafaunal species (e.g. giant sloths in the Americas or large marsupials in Australia), whereas others retained a greater diversity of native herbivores and predators [8]. Climatic warming, occurring simultaneously, further complicated these spatial patterns [9]. Moreover, domesticates spread unevenly, with pastoral and agricultural economies arising at different times across Eurasia, Africa and the Americas [10]. As a result, faunal communities that once showed distinct geographic patterns could have converged in composition if domestic species spread widely, or they might have become more fragmented in composition if local factors took precedence.

Despite many case studies, the global-scale reorganization of mammal communities across this transition remains largely underexplored [11]. Previous work has typically focused on single continents or specific taxonomic groups [6,12,13], leaving open the question of whether the Holocene, once domesticates entered the scene, ushered in fundamentally new community structures. Standard clustering methods often confuse geographic proximity with ecological similarity, obscuring whether Holocene assemblages maintained historical biogeographic boundaries or represented genuinely new groupings. Recent findings suggest that even small changes in species co-occurrences can ripple across entire ecosystems [14], implying that the replacement of wild fauna with domesticates might leave a large biogeographic footprint. Such findings imply that even limited changes, such as substituting wild fauna with domesticates, could significantly alter ecological relationships and community structure.

Here, we address this gap by examining a diverse suite of Pleistocene and Holocene fossil mammal assemblages from multiple continents and islands. We focus on presence–absence data to avoid the uneven biases of zooarchaeological counts (e.g. due to differential preservation or excavation methods). To uncover patterns, we apply two complementary clustering techniques: a spatially constrained hierarchical method [15] and a new unconstrained ‘chase clustering’ algorithm. The first approach anchors clusters partly in geographic distance, while the second relies only on compositional similarity, permitting regions to emerge or dissolve in unexpected ways. By comparing Late Pleistocene and Holocene groupings, we test whether domesticates and intensified land use disrupted earlier faunal boundaries. Our chase algorithm infers cluster number endogenously and, unlike agglomerative methods, imposes no geographic constraint—crucial for a global test of Holocene realignment.

We hypothesize that intensified land use and domestication amplified human impacts well beyond the megafaunal extinctions of the late Quaternary. We expect to see shifts in how sites group together, particularly in regions where livestock arrived early and spread widely. We extend prior continental studies to a global scale, testing whether departures from historical faunal boundaries provide evidence for agriculture’s role in reshaping mammal community structure.

## 2. Material and methods

### (a) Study system and data collection

We sourced the zooarchaeological data on mammals assembled for the Ecological Register (http://ecoregister.org/?a=downloadForm), downloaded on 6 June 2023. These come from peer-reviewed sources and museum records, with a broad global coverage and a focus on sites with comprehensive counts of identifiable faunal remains (e.g. [3,16–19]). We prioritized studies with radiometric or stratigraphic dating to ensure robust chronological control. Papers reporting only a minimum number of individuals or vague specimen tallies were excluded, as were poorly dated sites. Despite these efforts, Holocene sites largely represent human-generated assemblages (e.g. middens), potentially underestimating species richness. However, because our primary goal was to investigate biogeographic patterns rather than raw diversity, this limitation does not undermine our core questions. We screened all published site inventories but retained only those with exact taxon-level number of identified specimens and secure dating. This yielded a Pleistocene dataset of 475 mammal species from 191 sites and a Holocene dataset of 350 species (including 12 domesticates) from 206 sites, representing the largest globally balanced dataset currently achievable.

We applied a minimum-unique-species rule by retaining only the most specific non-overlapping identifications, following reconciliation of synonymous taxa. Variables for analysis were species occupancy (presence/absence) at each site and geographical coordinates for each assemblage. Assemblage-formation modes (e.g., midden versus pitfall traps) chiefly affect recovery of small or fragile taxa; our focus on medium–large mammals inherently minimizes these biases. For further details, see electronic supplementary material, with site information provided in electronic supplementary material, table S1.

### (b) Data processing

Data from all sources were synthesized into a standardized format for subsequent analysis. For each site, we recorded latitude and longitude, and for this analysis, we reduced the inventory data to the presence–absence of each mammal species, after filtering out sites with fewer than five species to avoid unstable compositional signals. Final matrices for the Pleistocene and Holocene epochs had rows representing species and columns as sites. We also constructed a coordinate file indicating the precise location of each site in decimal degrees (WGS84 reference system) and computed geographic distances between sites from these coordinates.

### (c) Chase clustering algorithm

**Figure 1.**
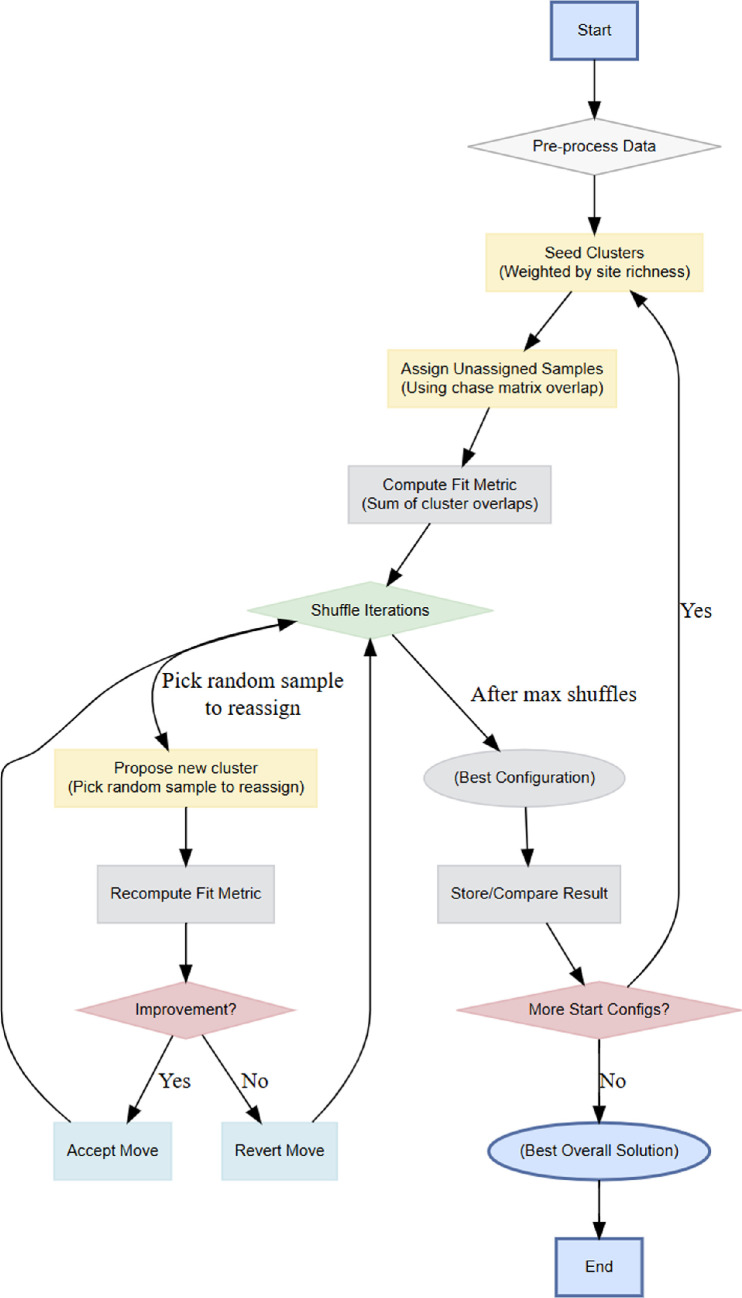
Schematic of the ‘chase clustering’ algorithm, which uses compositional overlap in ecological communities to identify and refine cluster assignments. First, sites with insufficient data are filtered out, then clusters are seeded from weighted samples. Unassigned sites join their most similar cluster. The algorithm shuffles assignments iteratively to maximize compositional coherence, retaining the best solution after multiple starts.

We developed a novel unconstrained ‘chase clustering’ method specifically to test whether spatial patterns in mammal communities emerge naturally from compositional similarity alone, unanchored explicitly to geography. The algorithm’s logic is illustrated in figure 1. Clusters were initiated (‘seeded’) using sites with distinct species compositions, prioritized according to their overall species richness. A ‘chase matrix’ was constructed from pairwise comparisons of species presence–absence across sites. In turn, remaining sites were ‘chased’ into the clusters they most resembled. We explored *γ* ∈ [0, −0.30] on the Pleistocene matrix. As γ became more negative, the number of clusters (*k*) increased from 2 (*γ* = 0) to 14 (*γ* = −0.3). A value of *γ* = −0.10 produced six compositionally coherent, geographically interpretable clusters without over-fragmentation (see Results). We therefore adopted *γ* = −0.10 for all subsequent analyses. With *γ* fixed, we re-ran chase clustering for the Holocene, again searching *k* = 2−15 (250 random starts, 10 000 shuffles each for stability), in a manner reminiscent of simulated annealing [20]. The algorithm imposes a heavy fit penalty on ‘clusters’ that include only a single site. By also using a Pleistocene-inherited *γ* regularization parameter for the Holocene, we allow solely the data to determine what *k* emerges for that epoch. For further details on the algorithm, see electronic supplementary material.

### (d) Constrained hierarchical clustering

For a comparison, we also applied a modified version of Ward’s hierarchical clustering [21] that integrates both compositional and geographic information [15]. Unlike chase clustering, the hierarchical approach requires specifying the number of clusters beforehand. This method computes two dissimilarity matrices: one based on species occupancy (Euclidean distance), and a second derived from great-circle distances among sites. A mixing parameter, alpha (*α*), then weights these matrices in a convex combination. Low *α* values emphasize composition; higher values emphasize geographic proximity. The approach iteratively merges sites to minimize within-cluster variance across both matrices. This was implemented using hclustgeo (ClustGeo package) with the number of clusters set to the chase-clustering result. Specifically, chase yielded *k* = 6 on the Pleistocene data, and we fixed that same *k* for Ward in both epochs to ensure a like-for-like comparison without further tuning. To select *α*, we ran increments of 0.01 between 0 and 1 and chose the value maximizing variance explanation (inertia). The resulting dendrogram was partitioned into clusters matching the number identified via chase clustering to compare global patterns between the two epochs.

### (e) Composition of wild versus domesticate species

For our main Holocene comparison, we excluded all domesticated taxa to focus on shifts within wild mammalian communities. We identified domesticates using established zooarchaeological criteria [18], removing them from the Holocene dataset to create a matrix directly comparable to the Pleistocene. This dataset is referred to as Holocene Wild. We then did a separate analysis using the full matrix (including domesticates, termed Holocene All) to measure how much these newly introduced species altered cluster structure, by comparing site assignments using the adjusted Rand index (ARI) [22]. This highlighted the extent to which domesticates reshaped Holocene biogeographic patterns beyond the changes observed in wild fauna.

### (f) Turnover from Pleistocene to Holocene

For 34 locations with paired Late Pleistocene and all Holocene records, we measured turnover directly at approximately the same locality (i.e., grouped sites within a 50 km radius). Turnover was quantified using the Jaccard distance, calculated as species gained or lost between epochs divided by the total species observed across both periods [23]. We then mapped these turnover values to visualize which regions exhibited the greatest compositional shifts over time.

### (g) Implementation and reproducibility

All analyses were conducted using R v. 4.4.2 [24], employing packages dplyr for data manipulation, ggplot2 for visualization and ClustGeo for constrained clustering. Custom R functions were written for chase clustering.

The R scripts for data import, filtering, clustering and visualization, and data are provided on Zenodo [25], ensuring reproducibility. A detailed description of chase clustering and a comparison with Ward’s geographically constrained hierarchical approach is given in the electronic supplementary material.

## 3. Results

We tested the regularization parameter (*γ*), ranging from 0 to −0.20, to fine-tune the chase clustering for the Pleistocene data. At *γ* = 0, only two clusters emerged (Australia versus the rest of the world), whereas *γ* = −0.05 yielded four broad continental groupings. Further decreases in *γ* continued isolating unique regional sites (e.g. southern South America, New Guinea). At *γ* = −0.10, six coherent clusters captured major continental differences while avoiding excessive fragmentation. Beyond *γ* = −0.15, solutions became highly subdivided (up to 12 clusters). We therefore selected *γ* = −0.10 as it produced a stable partition with meaningful biogeographic structure for the Pleistocene and then also used this *γ* value for the Holocene analysis.

**Figure 2.**
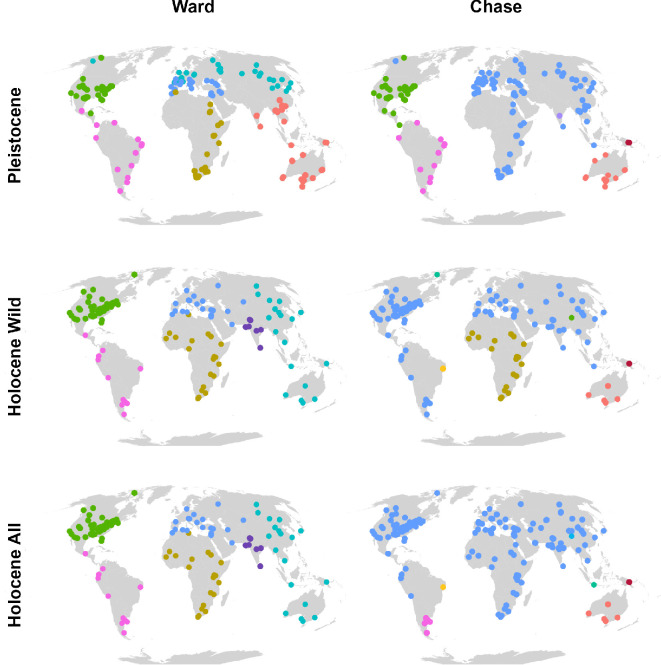
Late Quaternary mammal site clusters. Left column: Ward’s geography-weighted clustering (*α* = 0.31). Right column: composition-only chase clustering (*γ* = −0.10). Rows show datasets: Late Pleistocene (top), Holocene Wild taxa only (middle), Holocene Wild + domesticates (bottom). Colours denote cluster IDs within each row (IDs differ between rows). Ward’s six largely continental clusters change little across epochs, reflecting its spatial constraint. Chase clusters are similar to Ward in the Pleistocene but realign in the Holocene, especially when domesticates are included, highlighting compositional turnover that is not driven by geographic proximity.

Figure 2 contrasts Ward’s geography-weighted and chase composition-weighted solutions; colours are cluster IDs unique to each row (figure 2). Ward clustering consistently distinguishes the Americas, whereas Africa remains grouped closely with Eurasia. Australia and Southeast Asia are separate from the Palaearctic in the Pleistocene, but they unify more in the Holocene, and Europe exhibits greater internal partitioning during the Pleistocene. Notably, the Ward clusters do not change when domesticates are included (Holocene Wild versus All). By contrast, chase clustering yields partially overlapping but more flexible partitions. In the Pleistocene, the Americas again stand apart, while Africa, Europe and Asia form a broad ensemble, with distinctive smaller clusters highlighting unique regional faunas in India, New Guinea and Australia. In the Holocene Wild dataset, Africa detaches from Eurasia, and North–South American sites show more similarity. Inclusion of domesticates (Holocene All) further reshapes clusters, uniting some previously separate regions (e.g., Africa) but also creating highly localized clusters (e.g., in Brazil and Java), highlighting how domestication homogenizes some regions yet fragments others. The proportion of domesticated species relative to the site’s total richness is shown in electronic supplementary material, figure S1.

Of the 350 Holocene species in our dataset, 12 were domesticated mammals, appearing in roughly half of the global sites (110 of 206, with a mean of 1.47 domesticates per site). Comparing chase clustering solutions generated from only wild species versus those generated from all species (i.e., including domesticates) yielded an ARI = 0.433. While this indicates moderate concordance, it clearly shows that domesticates significantly restructured Holocene community patterns.

**Figure 3.**
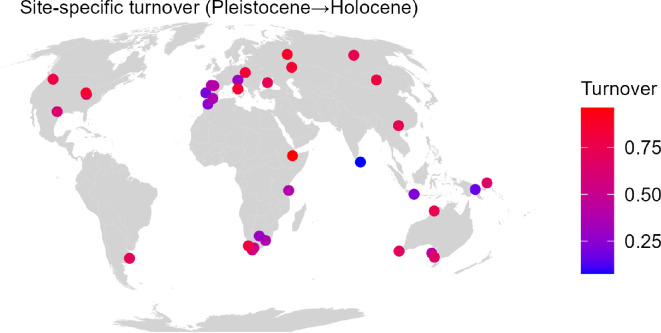
Regional‐level faunal turnover from the Late Pleistocene to the Holocene at 34 multi‐site locations. Domesticated species are included in Holocene locations. Circles represent proportional species turnover (losses or gains); warmer colours (red) indicate higher turnover, and cooler colours (blue/purple) indicate lower turnover. The variability highlights regionally distinct responses to human and environmental pressures.

Across the 34 aggregated (multi-site) localities common to both epochs, turnover from Pleistocene to Holocene varied considerably (figure 3). Regions such as New Guinea and Sri Lanka experienced minimal turnover, whereas western Europe and eastern Africa exhibited moderate levels of turnover. In contrast, the Americas, Australia, eastern Europe and parts of Asia, including Ethiopia and southern Africa, experienced the highest turnover. This geographic heterogeneity suggests that anthropogenic (e.g., farming, intensified land use) and environmental drivers (post-glacial climate change) affected mammal communities unevenly across the globe.

## 4. Discussion

Our modelling revealed that Holocene mammal communities diverged in several important ways from those of the Late Pleistocene, underscoring regionally variable impacts of farming and livestock introductions. Clusters in the Pleistocene data often aligned with broad continental regions, consistent with climatic gradients and physical barriers such as mountain ranges and oceans [7,8]. In contrast, Holocene groupings showed fluid boundaries under unconstrained chase clustering, indicating that domestic animals and other human impacts disrupted faunal communities beyond climate-driven expectations.

Including domesticated species strongly influenced chase cluster assignments. Domesticates spread widely, creating new compositional similarities among geographically distant regions—for instance, mammal communities in Europe and Africa converged due to the adoption of domesticated species originating in the Middle East [4,10]. When we removed domesticates from Holocene analyses, some regions regained a resemblance to Pleistocene patterns, implying that a subset of introduced taxa had an outsized role in determining site similarity. This finding aligns with Tóth *et al.* [14], who showed that even moderate changes in species interactions can reshape macro-scale co-occurrence patterns. Domesticates spread across multiple continents, creating new overlaps among sites separated by large distances. This effect would remain hidden in methods that assume strictly contiguous ranges or that place strong emphasis on geographical adjacency. Spatially constrained Ward’s clustering misses these effects, illustrating how anthropogenic factors can transcend traditional biogeographic boundaries, as well as showing the utility of the new chase algorithm in detecting non-spatially contiguous similarities.

At the same time, these transformations varied by region. Turnover analyses identified stable regions (e.g. Oceania) and others undergoing significant shifts (e.g., the Americas, Eurasia). Differences in the timing and intensity of agriculture may explain some of this heterogeneity, but other factors, like pre-existing environmental conditions or local cultural practices, also likely mattered [11,12]. In the Americas, substantial Late Pleistocene megafaunal losses were followed by regional domestication of native mammals, notably llamas and alpacas in South America, reshaping faunal communities long before European livestock introductions [13,26]. Elsewhere, indigenous mammalian domesticates (e.g. cattle, sheep, pigs) spread unevenly, producing patchier community compositions.

Despite strong domestication signals, our analysis has inherent limitations. Site-occupancy data can hide abundance shifts, and zooarchaeological assemblages may be skewed by human food preferences or differential bone preservation. Yet the coherent clustering of Holocene African faunas (even after domestic species are removed) indicates that our patterns are not simply artefacts of midden bias [8]. We therefore treat the dataset as a broad compositional map, suitable for global inference but not for local population dynamics. Across repeated random-start chase runs, over 80% of sites consistently joined the same clusters, demonstrating solution stability. However, a future step might include formal ensemble averaging, along with analyses of abundance metrics, environmental proxies and additional taxa (e.g., birds, plants), to further clarify drivers of species turnover.

Broadly, these findings underscore how human-driven extinctions, agriculture and resource extraction profoundly reshaped mammal community structures [2,12]. Modern biogeographic patterns have deep historical roots shaped by ancestral choices about species cultivation, trade and conservation [3]. Such perspective may guide current conservation efforts by reminding us that ecosystems are neither fixed nor solely climate-driven but reflect ongoing interactions between people and the rest of the biosphere. How we manage these interactions today will determine whether mammal communities become resilient or increasingly destabilized.

## Data Availability

All data and R code required for full replication are provided at https://github.com/bwbrook/chase-clustering, archived and citable via Zenodo [25]. J.A. devised the chase-clustering algorithm; B.W.B. implemented the R function. Supplementary material is available online [27].

## References

[1] Smil V. 2011 Harvesting the biosphere: the human impact. Popul. Dev. Rev. **37**, 613–636. (10.1111/j.1728-4457.2011.00450.x) 22319767

[2] Ellis EC *et al*. 2021 People have shaped most of terrestrial nature for at least 12,000 years. Proc. Natl Acad. Sci. USA **118**, e2023483118. (10.1073/pnas.2023483118) 33875599 PMC8092386

[3] Stephens L *et al*. 2019 Archaeological assessment reveals Earth’s early transformation through land use. Science **365**, 897–902. (10.1126/science.aax1192) 31467217

[4] Ahmad HI, Ahmad MJ, Jabbir F, Ahmar S, Ahmad N, Elokil AA, Chen J. 2020 The domestication makeup: evolution, survival, and challenges. Front. Ecol. Evol. **8**, e103. (10.3389/fevo.2020.00103)

[5] Cooke R *et al*. 2022 Anthropogenic disruptions to longstanding patterns of trophic-size structure in vertebrates. Nat. Ecol. Evol. **6**, 684–692. (10.1038/s41559-022-01726-x) 35449460

[6] Smith FA, Elliott Smith EA, Villaseñor A, Tomé CP, Lyons SK, Newsome SD. 2022 Late Pleistocene megafauna extinction leads to missing pieces of ecological space in a North American mammal community. Proc. Natl Acad. Sci. USA **119**, e2115015119. (10.1073/pnas.2115015119) 36122233 PMC9522422

[7] Boivin NL, Zeder MA, Fuller DQ, Crowther A, Larson G, Erlandson JM, Denham T, Petraglia MD. 2016 Ecological consequences of human niche construction: examining long-term anthropogenic shaping of global species distributions. Proc. Natl Acad. Sci. USA **113**, 6388–6396. (10.1073/pnas.1525200113) 27274046 PMC4988612

[8] Turvey ST, Fritz SA. 2011 The ghosts of mammals past: biological and geographical patterns of global mammalian extinction across the Holocene. Phil. Trans. R. Soc. B **366**, 2564–2576. (10.1098/rstb.2011.0020) 21807737 PMC3138610

[9] Lyons SK, Miller JH, Fraser D, Smith FA, Boyer A, Lindsey E, Mychajliw AM. 2016 The changing role of mammal life histories in Late Quaternary extinction vulnerability on continents and islands. Biol. Lett. **12**, 20160342. (10.1098/rsbl.2016.0342) 27330176 PMC4938058

[10] Larson G, Fuller DQ. 2014 The evolution of animal domestication. Annu. Rev. Ecol. Evol. Syst. **45**, 115–136. (10.1146/annurev-ecolsys-110512-135813)

[11] Rueda M, González‐Suárez M, Revilla E. 2024 Global biogeographical regions reveal a signal of past human impacts. Ecography **2024**, e06762. (10.1111/ecog.06762)

[12] Hatfield JH, Davis KE, Thomas CD. 2022 Lost, gained, and regained functional and phylogenetic diversity of European mammals since 8000 years ago. Glob. Chang. Biol. **28**, 5283–5293. (10.1111/gcb.16316) 35748709 PMC9540530

[13] Lyons SK *et al*. 2016 Holocene shifts in the assembly of plant and animal communities implicate human impacts. Nature **529**, 80–83. (10.1038/nature16447) 26675730

[14] Tóth AB *et al*. 2019 Reorganization of surviving mammal communities after the end-Pleistocene megafaunal extinction. Science **365**, 1305–1308. (10.1126/science.aaw1605) 31604240

[15] Chavent M, Kuentz-Simonet V, Labenne A, Saracco J. 2018 ClustGeo: an R package for hierarchical clustering with spatial constraints. Comput. Stat. **33**, 1799–1822. (10.1007/s00180-018-0791-1)

[16] Ament JM, Carbone C, Crees JJ, Freeman R, Turvey ST. 2023 Anthropogenic predictors of varying Holocene occurrence for Europe’s large mammal fauna. Biol. Lett. **19**, 20220578. (10.1098/rsbl.2022.0578) 37073526 PMC10114012

[17] Davoli M, Kuemmerle T, Monsarrat S, Crees J, Cristiano A, Pacifici M, Svenning JC. 2024 Recent sociocultural changes reverse the long‐term trend of declining habitat availability for large wild mammals in Europe. Diver. Distribut **30**, e13921. (10.1111/ddi.13921)

[18] Larson G *et al*. 2014 Current perspectives and the future of domestication studies. Proc. Natl Acad. Sci. USA **111**, 6139–6146. (10.1073/pnas.1323964111) 24757054 PMC4035915

[19] Carter BE, Alroy J. 2024 Energy use of modern terrestrial large mammal communities mirrors Late Pleistocene megafaunal extinctions. Front. Biogeogr. **16**, e62724. (10.21425/f5fbg62724)

[20] Kirkpatrick S, Gelatt CD, Vecchi MP. 1983 Optimization by simulated annealing. Science **220**, 671–680. (10.1126/science.220.4598.671) 17813860

[21] Murtagh F, Legendre P. 2014 Ward’s hierarchical agglomerative clustering method: which algorithms implement ward’s criterion? J. Classif. **31**, 274–295. (10.1007/s00357-014-9161-z)

[22] Hubert L, Arabie P. 1985 Comparing partitions. J. Classif. **2**, 193–218. (10.1007/BF01908075)

[23] Jaccard P. 1901 Étude comparative de la distribution florale dans une portion des Alpes et du Jura. Bull. Soc. Vaudoise Sci. Nat. **37**, 547–579.

[24] R Core Team. 2025 R: a language and environment for statistical computing. Vienna, Austria: R Foundation for Statistical Computing. See https://www.R-project.org/.

[25] Brook BW. 2025 Data and code from: Late Pleistocene faunal community patterns disrupted by Holocene human impacts. Zenodo. (10.5281/zenodo.15515260)40795982

[26] Hedberg CP, Lyons SK, Smith FA. 2022 The hidden legacy of megafaunal extinction: loss of functional diversity and resilience over the Late Quaternary at Hall’s Cave. Glob. Ecol. Biogeogr. **31**, 294–307. (10.1111/geb.13428)

[27] Brook BW, Lyons SK, Carter BE, Gearty W, Todorov OS, Aandahl Z *et al*. 2025 Supplementary material from: Late Pleistocene faunal community patterns disrupted by Holocene human impacts. Figshare. (10.6084/m9.figshare.c.7960790)40795982

